# Esthetic Rehabilitation of Complicated Crown Fractures Utilizing Rapid Orthodontic Extrusion and Two Different Restoration Modalities

**DOI:** 10.5005/jp-journals-10005-1136

**Published:** 2012-02-24

**Authors:** Sladana Milardovic Ortolan, Mihovil Strujic, Andrej Aurer, Josko Viskic, Lana Bergman, Ketij Mehulic

**Affiliations:** Teaching Assistant, Department of Prosthodontics, School of Dental Medicine, University of Zagreb, Gunduliceva 5, 10000 Zagreb, Croatia e-mail: milardovic@sfzg.hr; Teaching Assistant, Department of Orthodontics, School of Dental Medicine, Zagreb, Croatia; Assistant Professor, Department of Periodontics, School of Dental Medicine, Zagreb, Croatia; Teaching Assistant, Department of Prosthodontics, School of Dental Medicine, Zagreb, Croatia; Teaching Assistant, Department of Prosthodontics, School of Dental Medicine, Zagreb, Croatia; Professor, Department of Prosthodontics, School of Dental Medicine Zagreb, Croatia

**Keywords:** Complicated tooth fracture, Orthodontic extrusion, Esthetic rehabilitation, Prosthodontic restoration, Direct adhesive restoration

## Abstract

This case report describes the management of a crown-root fractured maxillary right central incisor and a crown fractured maxillary left central incisor using two different techniques.

A complex procedure was designed to manage this case including orthodontic extrusion to move the fracture line above the alveolar bone and surgical recontouring of the altered gingival margin. Finally, the right incisor was restored prosthodontically. Prosthetic treatment was based on performing a post and core, and all-ceramic crown on the extruded tooth. The left, less-damaged incisor was restored directly using composite resin.

The treatment resulted in good esthetics and secured periodontal health. This case report demonstrates that a multidisciplinary treatment approach is a reliable and predictable option to save a tooth.

**How to cite this article:** Ortolan SM, Strujic M, Aurer A, Viskic J, Bergman L, Mehulic K. Esthetic Rehabilitation of Complicated Crown Fractures Utilizing Rapid Orthodontic Extrusion and Two Different Restoration Modalities. Int J Clin Pediatr Dent 2012;5(1):64-67.

## INTRODUCTION

Complicated crown fractures involve enamel, dentine and the pulp, whereby the most commonly affected tooth is the maxillary central incisor. Fractures of anterior teeth cause not only esthetic and functional, but also psychological problems. Various treatment modalities are available depending upon the clinical, physiological and radiographic status of the involved tooth. In complex cases, a combination of endodontic, periodontal, orthodontic and restorative procedures may be required.^1^

The first step in treatment is to expose sound supragingival tooth structure to allow restoration. Procedures like crown lengthening, surgical extrusion or orthodontic extrusion are possible options. Orthodontic extrusion is a biological way to expose sound tooth structure,^2,3^ but it usually requires prolonged treatment. Rapid coronal movement with intense force provides faster clinical results.^4^

In this case report, a multidisciplinary treatment of complicated fractures of both maxillary central incisors in a 12-year-old boy is presented.

## CASE REPORT

A 12-year-old boy was referred to the Clinic of Dental Medicine in Zagreb 2 months after a sports accident which resulted in fracture of upper anterior teeth. The parents and the patient wished for an esthetic, metal-free restoration. Clinical examination revealed an oblique crown-root fracture of tooth 11 with the fracture line extending subgingivally at the mesiopalatal aspect (Figs 1A and B). Tooth 21 showed a crown fracture in the coronal third. Both teeth were previously endodontically treated and provisionally restored alio loco. Radiographic examination confirmed clinical findings. A definitive treatment plan was designed as follows. Two restoration modalities should be performed. The left, dominantly preserved incisor should be restored conservatively with composite resin, whereby for the right incisor an all-ceramic crown was planned. Therefore, it was necessary to extrude the tooth in the first phase to allow sufficient tooth area available for core build- up and crown preparation. Edgewise brackets were bonded to teeth in the intercanine segment, whereby the bracket was placed more gingivally on the tooth to be extruded. Extrusive force was provided by an orthodontic wire (Fig. 2). After 3 weeks, around 3.5 mm of extrusion had been achieved followed by 8 weeks of retention period. Afterward periodontal plastic surgery was performed to correct the discrepancy of the gingival margin due to coronal migration of the connective tissue (Figs 3 and 4). In the next phase, post and core build-up was performed (FRC Postec and Multicore Flow, Ivoclar Vivadent, Schaan, Liechtenstein). A provisional crown was placed for 2 months to stabilize the soft tissue (Figs 5 and 6). In the last phase, the teeth were definitively restored. The left incisor was restored directly using composite material IPS Empress Direct (Ivoclar Vivadent) (Figs 7 to 9). On the right extruded incisor, an all-ceramic crown (IPS Empress II, Ivoclar Vivadent) was placed. Good esthetics was achieved (Fig. 10). The patient was kept on recall of 1, 3, 6 and 12 months. The periodontal tissues remained normal and neither luxation nor relapse was noted. The patient reported no problems.

**Fig. 1A:**
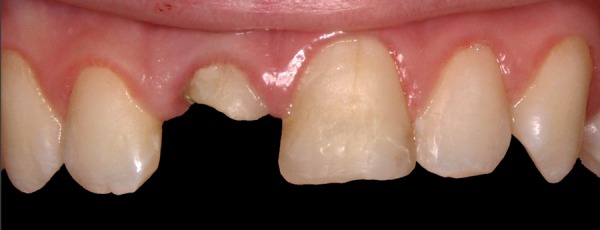
Preoperative appearance with provisional, esthetically unpleasant restoration on tooth 21 and glass-ionomer cement on 21

**Fig. 1B:**
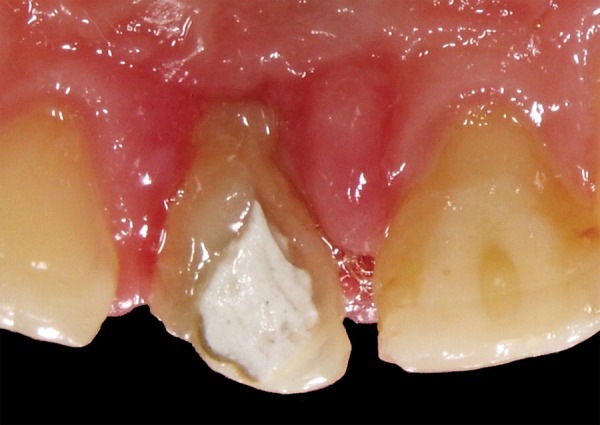
Palatal view

**Fig. 2:**
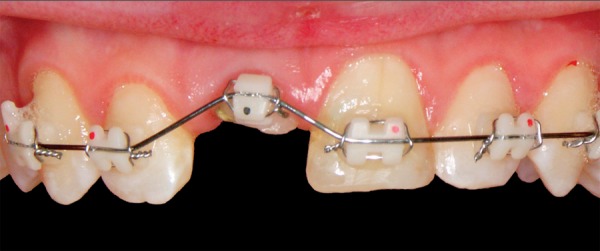
Orthodontic extrusion—edgewise brackets in the intercanine segment

**Fig. 3:**
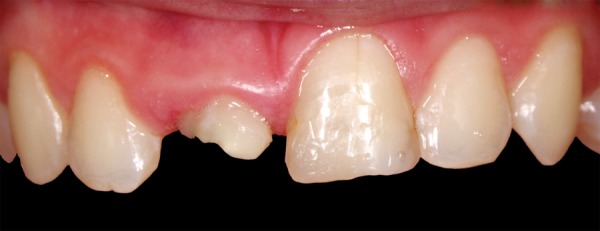
After 3 weeks, around 3.5 mm of extrusion had been achieved. Coronal migration of soft tissue is notable

**Fig. 4:**
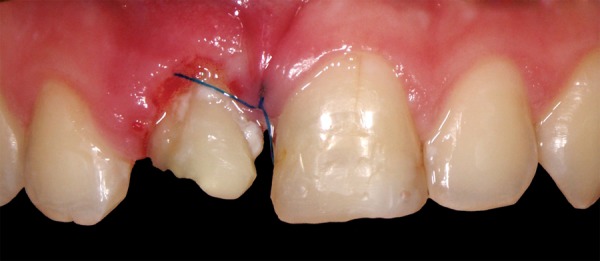
Situation immediately after periodontal plastic surgery

**Fig. 5:**
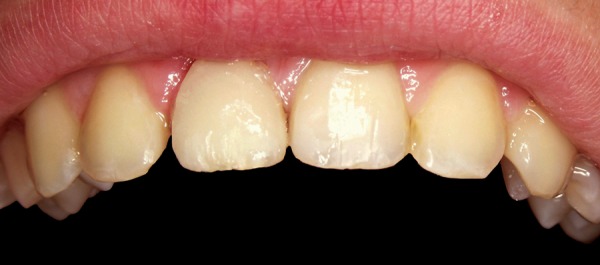
Placement of a provisional acrylic crown for 2 months on tooth 11 to stabilize the soft tissue

**Fig. 6:**
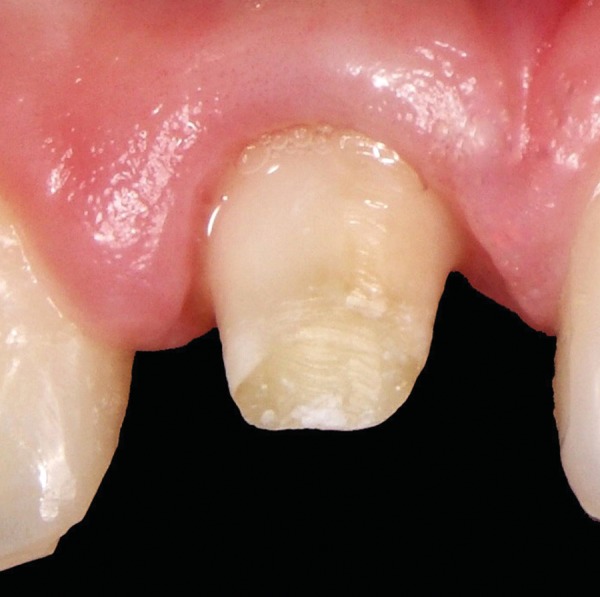
Appearance after 4 weeks of soft tissue stabilization

**Fig. 7:**
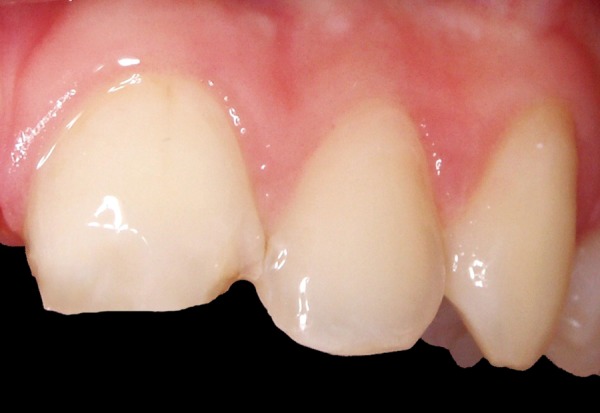
Tooth 21 after removal of provisional composite restoration

## DISCUSSION

Crown-root fractures are usually a result of facial injuries which may occur during sport activities, car accidents, falls or fights. This kind of injury mainly affects children and adolescents, with boys considered as being at a higher risk.^5^The emotional trauma as consequence of tooth loss/demage with consequence of being different during the sensitive age should not be underestimated. It is extremely important to put all efforts in preventing our patient from this emotional trauma.

**Fig. 8:**
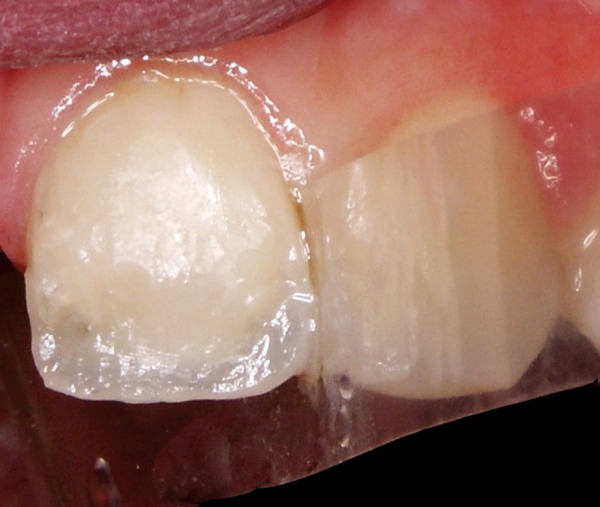
Direct composite restoration with layering technique (IPS Empress Direct, Ivoclar Vivadent, Schaan, Liechtenstein)

**Fig. 9:**
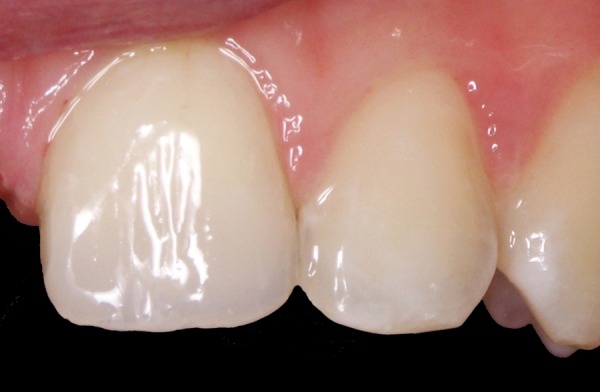
Direct composite restoration—final result

**Fig. 10:**
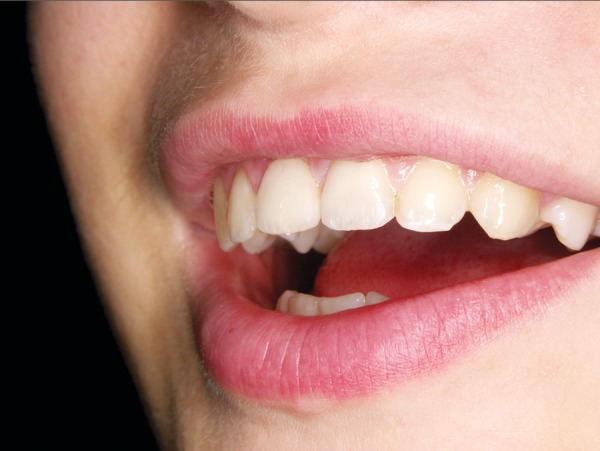
The definitive smile was created with an all-ceramic crown on tooth 11 (IPS Empress II, Ivoclar Vivadent, Schaan, Liechtenstein) and a direct composite restoration on tooth 21

There are several methods of treatment depending on the situation. Placement of an endodontic post is indicated in situations when there is no less than 1 mm of healthy tooth structure supragingivally. If the fracture line is positioned below gingival-free margin, and if the length of the apical root is sufficient enough to support a coronal restoration, then the root can be orthodontically extruded to elevate the fracture plane above the gingival margin and allow for a ferrule effect.^6-9^

Continuous light forces have been recommended for slow orthodontic extrusion. However, this may cause reverse osseous architecture around the tooth being extruded.^10^ In case of rapid extrusion, the periodontal fibers stretch and readjust, but the bone does not have time to remodel because of rapid movement. Thus, there is no coronal shift of the marginal bone, facilitating prosthetic restoration as there is no need to reshape bone.^4,11^

However, coronal displacement of the gingival margin can be stimulated under extrusive force. To prevent this phenomenon, some authors indicate supracrestal fiberotomy during the active extrusion period.^12,13^ Berglundh et al demonstrated that repeated fiberotomy led to pronounced recession of the gingival margin and extensive loss of the connective tissue attachment.^14^ Furthermore, facial fiberotomy would result in buccal recession.^14^ In the present case, rapid orthodontic extrusion was not associated with fiberotomy because surgical recontouring of the altered gingival margin prior to tooth restoration is considered to be simple, minimally invasive and reliable.^15^

Prior to final restoration, it is advisable to retain the root in its new position to prevent relapse.^10,11^ Additional few weeks are necessary for tissue maturation after periodontal surgery, during which period a provisional acrylic crown can be placed. This points out the main disadvantages of this technique: Relatively long time of treatment, long period of stabilization, and the need to use an orthodontic appliance which is not always convenient for the patient, not to forget poor esthetics during treatment. That is why this technique requires both commitment and motivation from the patient and the dentist.

In the past, fractured teeth were restored using acrylic resin or PFM restorations. These restorations did not promote adequate esthetics. Recently, with the advancement in the materials and bonding techniques, various options have been suggested to achieve the desired goal. Nowadays it is possible to combine prosthodontic procedures using all-ceramic restorations with a minimally invasive approach using new composite materials and ensure excellent esthetics. In the presented case, two different techniques used have met with good success.

## CONCLUSION

After rapid orthodontic extrusion, satisfactory esthetics was achieved by applying two different restoration methods, the direct with composite material, and the indirect using a glass- ceramic crown.
